# Iodoform

**Published:** 1884-08

**Authors:** A. J. Reinhold

**Affiliations:** 6 West 125th Street; New York


### IODOFORM.

*Editor Independent Practitioner:*

I think it is now generally conceded by the members of our profession, that Iodoform is one of the best antiseptics at present in use. Its presence in roots of teeth from which putrescent pulps have been removed, and which for obvious reasons cannot always readily be made aseptic, is looked upon as a "friend at court."

Gutta-percha in chloroform is considered by very many successful men a reliable root filling, and so, being a believer in both iodoform and gutta-percha solution, I combine the two by making a saturated solution of iodoform and chloroform, and then adding gutta-percha enough to make it of the proper cream-like consistency for root filling.

With this combination in roots of teeth, such as I mentioned, I feel a certain security that I never have with any other material.

Cordially yours,

A. J. Reinhold,

New York, July 17, 1884. 6 West 125th Street.

